# The impact of accreditation of primary healthcare centers: successes, challenges and policy implications as perceived by healthcare providers and directors in Lebanon

**DOI:** 10.1186/1472-6963-14-86

**Published:** 2014-02-25

**Authors:** Fadi El-Jardali, Randa Hemadeh, Maha Jaafar, Lucie Sagherian, Ranime El-Skaff, Reem Mdeihly, Diana Jamal, Nour Ataya

**Affiliations:** 1 Department of Health Management and Policy, Faculty of Health Sciences, American University of Beirut, Riad El Solh, Beirut 1107 2020, Lebanon; 2 Ministry of Public Health, Beirut, Lebanon

**Keywords:** Accreditation, Primary healthcare, Healthcare providers, Lebanon

## Abstract

**Background:**

In 2009, the Lebanese Ministry of Public Health (MOPH) launched the Primary Healthcare (PHC) accreditation program to improve quality across the continuum of care. The MOPH, with the support of Accreditation Canada, conducted the accreditation survey in 25 PHC centers in 2012. This paper aims to gain a better understanding of the impact of accreditation on quality of care as perceived by PHC staff members and directors; how accreditation affected staff and patient satisfaction; key enablers, challenges and strategies to improve implementation of accreditation in PHC.

**Methods:**

The study was conducted in 25 PHC centers using a cross-sectional mixed methods approach; all staff members were surveyed using a self-administered questionnaire whereas semi-structured interviews were conducted with directors.

**Results:**

The scales measuring Management and Leadership had the highest mean score followed by Accreditation Impact, Human Resource Utilization, and Customer Satisfaction. Regression analysis showed that Strategic Quality Planning, Customer Satisfaction and Staff Involvement were associated with a perception of higher Quality Results. Directors emphasized the benefits of accreditation with regards to documentation, reinforcement of quality standards, strengthened relationships between PHC centers and multiple stakeholders and improved staff and patient satisfaction. Challenges encountered included limited financial resources, poor infrastructure, and staff shortages.

**Conclusions:**

To better respond to population health needs, accreditation is an important first step towards improving the quality of PHC delivery arrangement system. While there is a need to expand the implementation of accreditation to cover all PHC centers in Lebanon, considerations should be given to strengthening their financial arrangements as well.

## Background

A responsive and comprehensive primary healthcare (PHC) system leads to a more efficient health system, lower rates of hospitalization, fewer health inequalities, better health outcomes and lower costs [1-3]. Despite the integral role of PHC for health systems, the World Health Report (2008) indicated that countries “are not performing as well as they could and as they should” when it comes to PHC [4]. A major challenge hindering countries from delivering PHC is establishing and maintaining high quality services [5].

One increasingly employed method for promoting quality at the healthcare organizational level is accreditation [5,6]. Despite its routine use in the work course of most hospitals worldwide, accreditation has only recently has been introduced into the PHC setting in high- income countries (HICs) [7]. This recent emphasis on accreditation in PHC organizations came with the shift in healthcare policy from hospitals towards preventive and primary healthcare delivery services [8].

Accreditation of PHC practices was reported to increase emphasis on the role of PHC within the healthcare system and to ensure quality control and improvement [9]. A review of the literature about PHC accreditation by O'Beirne et al. identified a cross-sectional study by Braun et al. (2008) that revealed that accredited centers were more likely to have staff dedicated to risk management, environmental safety and quality improvement [10]. Also, accredited centers reported more quality assurance projects than non-accredited centers, more frequently audited their clinical records, used credentialing methods, reviewed providers and trained staff [10]. The literature review also showed that PHC accreditation results in improved teamwork, improved access to care, increased awareness of patient safety, improved practice systems and care processes and improved quality of care [10].

In the Eastern Mediterranean Region (EMR), an expert group meeting took place in Cairo in 2002 to discuss the implementation of accreditation [11]. The meeting concluded that although accreditation of health facilities is desired in countries of the EMR, the system required for implementing accreditation is not yet developed. In order to improve care through accreditation, it is important to establish leadership commitment and regulations to implement accreditation, allocate adequate resources, ensure the availability of data and facilitate its use [11].

Lebanon was the first low- and middle- income country (LMIC) in the EMR to develop and implement national hospital accreditation standards in 2002 [12,13]. Building on the successful implementation of hospital accreditation, the Lebanese Ministry of Public Health (MOPH) sought to strengthen its leadership and governance functions and to improve the quality of services through implementing a national accreditation system at PHC centers.

In Lebanon, most PHC is mainly provided in health centers and dispensaries. In 1996, a comprehensive assessment of health centers and dispensaries was conducted in Lebanon to identify those able to provide a minimal package of PHC services. Among more than 800 facilities, 29 PHC centers were selected to form the nucleus of a National Network [14]. This National Network has expanded to encompass 150 centers in 2012. Although several PHC centers are owned and managed by the MOPH or the Ministry of Social Affairs (MOSA), the majority are owned and managed by Non-Governmental Organizations (NGOs) [14].

The ministry’s contracting transactions with the centers does not involve financial transactions. This approach focused on encouraging NGOs to improve the community health status while benefiting from the ministry’s assistance in medical, paramedical and management training, and also in providing vaccines, essential drugs and medical and educational supplies [14]. The ministry has also assisted PHC centers in their quality improvement initiatives and efforts. The first national PHC strategy was developed in 1994 by the MOPH [15]. This strategy was updated in 2005 and in 2011 and stressed on the importance of implementing quality assurance procedures and the continuous improvement of services [15].

In 2009, the MOPH initiated the PHC accreditation program in collaboration with Accreditation Canada International (ACI) [16]. With the support of an expert working group in Lebanon, in 2010 ACI developed and piloted PHC accreditation standards. Starting April 2011, key stakeholders were trained in the accreditation process [17]. Pilot organizations then conducted a self-assessment (June to September 2011), the results of which were used to evaluate and refine standards [18]. The accreditation survey immediately followed self-assessment (October 2011 to June 2012); the surveys spanned two full days at the PHC centers and were conducted by external surveyors from ACI. This was the first accreditation survey targeting PHC centers in Lebanon.

Findings from a baseline assessment of PHC centers in Lebanon to implement accreditation standards revealed that PHC centers were at the early stages of preparation for accreditation. They lacked quality improvement plans and did not regularly review evidence-based guidelines or identify and monitor outcome measures or indicators. Moreover, most centers lacked a system for incident and accident reporting and did not complete a summary of care provision in the client’s record [16].

Given this lag in quality regulations and capacity at PHC centers in Lebanon, an incremental approach to implementing accreditation standards was undertaken starting with implementing accreditation in 25 centers in 2012, followed by evaluation and refinement of the process, and then scale-up. These 25 PHC centers were selected based on their large size, coverage as well as the representation of all the PHC centers in the country with regards to the services they provide and their distribution across all the geographic regions. These centers were selected as being representative of the 150 PHC centers in the MOPH network by the national working group on this project which included key stakeholders from MOPH and ACI. The 150 centers in the National PHC Network in Lebanon are part of the 1,085 PHC centers and dispensaries distributed across Lebanon. Most of the 1,085 centers are located in Mount Lebanon (402 centers; 37.1%), 219 (20.2%) centers are located in the South, 152 (14%) in Bekaa, 136 (12.5%) in Beirut, 113 (10.4%) in Nabatiyeh and only 63 (5.8%) in the North. The 150 centers in the national network are distributed across regions as follows: 34% in Beirut and Mount Lebanon, 29% in the South, 30% in the North and 17% in Bekaa [14]. All the centers provide the same services but technological differences exist between them [19]. They are managed by physicians, nurses and allied health professionals. The centers provide the following services, general medical care, pediatrics, dental and oral health, reproductive health, and cardiovascular medical care. In addition to those services, they play a major role in dispersing essential drugs. The MOPH is the main provider of the drugs distributed at these centers [19]. Irrespective of location, any health center that provides the above mentioned services package qualifies it to be called a PHC center [19].

This paper aims to gain a better understanding of (1) the impact of accreditation on quality of care as perceived by PHC staff members and directors; (2) how accreditation affected staff and patient satisfaction; (3) the key enablers and challenges to the implementation of accreditation; and (4) the possible strategies to improve implementation of accreditation in PHC.

## Methods

This study was conducted in 2012; several months after the centers conducted the accreditation survey. The study targeted all PHC staff and utilized a cross-sectional mixed methods approach composed of both a quantitative and a qualitative component.

The quantitative component employed a self-administered questionnaire adapted from a tool used by El-Jardali et al. (2008) [13], with minor changes to the wording to reflect primary healthcare. This tool was previously used in Lebanon to assess the perceived impact of accreditation on quality of care through the lens of healthcare professionals. It is composed of five sections covering quality of care, accreditation impact, a retrospective assessment of the accreditation process, awareness of the accreditation process and a section for demographic information (including age, gender, occupation and years of experience). The questionnaire included seven scales (composed of several items), these are: Management and Leadership (nine items), Strategic Quality Planning (seven items), Human Resource Utilization (six items), Quality Management (six items), Quality Results (five items), Customer Satisfaction (seven items) and Accreditation. The Accreditation scale is divided into three subscales which are Accreditation Impact (14 items), Staff Involvement in Accreditation (22 items) and Awareness of Accreditation (five items). The scale on Accreditation was adapted from Milner et al. (2007) [20] and was added to the tool used by El-Jardali et al. (2008) [13] for the purpose of this study. The survey was originally developed in English but translated to Arabic since that is the language respondents are most comfortable in. The survey was translated to Arabic by a professional translator. Two members of the research team conducted back translation to ensure that the correct wording and phrasing of questions is used throughout the survey. Cognitive interviewing [21,22] was conducted in lieu of a pilot with selected professionals at the MOPH to ensure the clarity of the questions and make additional corrections. Minor modifications to the wording of some questions were made to some questions mainly to ensure clarity of Arabic terms.

The qualitative component of the study consisted of semi-structured interviews with facility directors. The interview tool covered the following main topics: benefits of accreditation, the effect of accreditation on staff and patient satisfaction, enabling and success factors, and challenges to the process of accreditation as well as strategies for improving implementation of accreditation. The specific questions asked during the interview were:

1. Based on your experience, how has the accreditation process contributed to the improvement of the quality of care delivered by this center?

2. In your opinion, how sustainable are the changes brought about by accreditation?

3. May you share your views on how accreditation has affected your satisfaction as an employee?

4. To what extent do you think the accreditation process has affected patient satisfaction in this center?

5. List the top three barriers/challenges that you have faced throughout the accreditation process

6. What are, in your opinion, some strategies to better implement accreditation in the future?

### Sampling method

All 25 centers that conducted the accreditation survey participated in this study. These 25 centers are representative of the 150 PHC centers forming the PHC network in Lebanon. A total of 20 centers participated in both components of the study, three centers participated in the survey only, and two centers participated in the semi-structured interviews only. Centers that chose not to complete the survey or interview were not pressured into participation as that would breach our ethical protocol. The centers were distributed across all the five governorates in Lebanon (four centers in Beirut, seven in the South, two in the Bekaa, seven in Mount Lebanon, and five in the North). All staff members participated in the survey and facility directors participated in the semi-structured interviews. Table 1 presents total number of participating centers and response rates to the questionnaire.

**Table 1:** Questionnaire distribution and response rates

**Region**	**Number of centers per region**	**Number of recruited staff members per region**	**Number of respondents per region**	**Percent response rate per region**
Beirut	4	78	59	76%
South	7	112	75	67%
Bekaa	2	20	16	80%
Mount Lebanon	5	94	72	77%
North	5	99	85	86%
Total	23	403	307	76%

### Data collection

Ethical approval for the study was granted by the Institutional Review Board at the American University of Beirut. Written informed consent was collected from all participants prior to data collection

Facility directors were contacted by phone to request their participation. Upon their approval, the surveys were distributed to PHC staff members. Staff were assured that their participation was voluntary, their choice to participate would not affect their employment and that directors would not view their responses. Participants were requested to complete the survey during their free time and in a setting of their choice and to return it in a sealed envelope within one week of receiving it.

Interviews were digitally voice recorded after securing interviewee consent. Interviews were conducted in Arabic and transcribed verbatim immediately thereafter. Transcripts were then translated to English to facilitate data analysis. These English translated transcripts were reviewed by two members of the research team to ensure their validity.

### Data analysis

Data generated from the questionnaires was coded, entered, and analyzed using SPSS 20.0 at a significance level of 0.05. Univariate analysis was conducted to explore demographic characteristics of respondents. Cronbach’s Alpha was used to measure the internal consistency and reliability of the scales and all values were above 0.80 thus indicating good internal consistency and reliability. Scores were created by summation of the items within the scales and dividing by the number of items with non-missing values. This produced a score that varies between 1 and 5 for each scale with higher scores indicating higher agreement.

A linear regression model was used to understand the association between quality results and independent variables. The independent variables included in this model were the scores for the scales measuring Management and Leadership, Strategic Quality Planning, Quality Management, Human Resource Utilization, Customer Satisfaction, and Accreditation including Accreditation Impact, Staff involvement, and Accreditation Awareness. The model was controlled for age, gender, experience and position of respondent.

Thematic analysis was used for the analysis of interviews. The findings were first coded and brought together in a spreadsheet to better manage the data. Open coding was conducted first, where findings were broken into chunks that relate to different concepts or ideas. Axial coding was then conducted, which involves organizing the emerging concepts into themes and sub-themes. Themes were pre-identified based on the study objectives and interview questions.

## Results

### Quantitative results

Twenty-three PHC centers out of the 25 centers participated in the quantitative component of the study. Of the 403 questionnaires that were distributed to the 23 PHC centers, 307 were returned complete (response rate of 76%) (Table 1). As observed in Table 2, most respondents were females (65.1%), between 30 to 45 years of age (50.5%), and have been working at their centers between 5 to 10 years (34.8%). A total of 27.5% of respondents were physicians and 18% were nurses (Table 2).

**Table 2:** Demographics

	**N**	**%**
**Gender**		
Male	102	34.9
Female	190	65.1
**Age (Years)**		
< 30	63	21.5
30 – 45	148	50.5
46 – 55	57	19.5
> 55	25	8.5
**Experience (Years)**		
< 5	84	28.1
5 – 10	104	34.8
10.1 – 15	48	16.1
>15	63	21.1
**Work type**		
Director of the center	12	4.2
Nurse	51	18.0
Physician	78	27.5
Pharmacist	14	4.9
Social worker	13	4.6
Unit assistant/Clerk/Secretary	17	6.0
Technician (e.g. EKG, Lab, Radiology)	30	10.6
Administration/Management	18	6.3
Other	51	18.0

The mean scores computed for the scales and subscales were all high. Management and Leadership had the highest mean score (4.28) followed by Accreditation Impact (4.27), Human Resource Utilization and Customer Satisfaction (both having a mean score of 4.24), Staff Involvement (4.23), Strategic Quality Planning and Quality Results (both having a mean score of 4.21), Accreditation Awareness (4.18) and finally Quality Management (4.02) (Table 3).

**Table 3:** Descriptive statistics and Cronbach’s Alpha on survey scales

**Scale**	**Items**	**Mean (SD)**	**Cronbach’s alpha**
Management and leadership	9	4.28 (0.46)	0.902
Strategic quality planning	7	4.21 (0.47)	0.829
Quality management	6	4.02 (0.60)	0.823
Human resource utilization	6	4.24 (0.53)	0.854
Quality results	5	4.21 (0.52)	0.818
Customer satisfaction	7	4.24 (0.55)	0.906
Accreditation			
Accreditation impact	14	4.27 (0.48)	0.936
Staff involvement	22	4.23 (0.52)	0.958
Accreditation awareness	5	4.18 (0.55)	0.845

More than 90% of respondents strongly agreed that leadership is the driving force behind quality improvement. Respondents agreed that senior executives have a clear vision for improving quality, participate in activities to improve quality of care and allocate available resources to improving quality. In the Strategic Quality Planning scale, more than 90% of respondents indicated that staff members and middle managers play a key role in setting priorities for quality improvement; while 83.9% indicated that patients’ expectations about quality play a key role in setting these priorities. With regards to Quality Management, more than 90% of respondents agreed that equipment and supplies are regularly checked and that services are thoroughly tested for quality before implementation. Additionally, they indicated that the center encourages them to keep records of quality problems through documentation (See Additional file 1).

When it comes to Human Resources Utilization, only 62.5% stated that they were rewarded and recognized for improving quality. In the scale of Quality Results, around 90% of respondents agreed that their centers are showing steady measurable quality improvements in the quality of customer satisfaction, administration, and quality of care, despite financial constraints. With regards to patient satisfaction, more than 90% of respondents agreed that their centers perform a good job in assessing current and future patient needs and resolving complaints (See Additional file 1).

Whereas more than 90% of respondents indicated that they were aware of the accreditation process, its aims and objectives and were committed to participate in it, only 70% indicated that patients were aware that accreditation was underway. Additionally, only 78.2% indicated receiving sufficient training and support to fulfill their accreditation responsibilities. Around 75% of respondents indicated receiving recognition from their work colleagues and 77.9% indicated receiving recognition from their line managers for their contribution to the accreditation process. Importantly, the majority agreed that accreditation is a worthwhile process (97.4%) and that it had a positive impact on the centers, including: increased responsiveness of centers when changes are to be implemented (94.6%), motivation of staff and team work (94.9%), development of collaboration partners in the health care system (92.6%), and improved standards and delivery of healthcare (91.9%) (See Additional file 1).

Results of the linear model indicated that the score on Quality Results increased by 0.297 (p-value = 0.003) for every unit increase in the score on Strategic Quality Planning. An increase of 0.412 (p-value = 0.008) in Quality Results was also observed for every unit increase in the score on Customer Satisfaction. Quality Results also increased by 0.309 (p-value = 0.004) for every unit increase in Staff Involvement in Accreditation (Table 4). Readers should be reminded that these findings are based on staff perceptions and not indicators that are continuously being measured at PHC centers.

**Table 4:** Linear regression results

	Beta* (Std. error)	**P-value**
Management and leadership	0.072 (0.084)	0.390
Strategic quality planning	0.297 (0.099)	***0.003*** **
Quality management	-0.079 (0.072)	0.274
Human resource utilization	0.073 (0.083)	0.378
Customer satisfaction	0.412 (0.063)	***<0.001*** **
Accreditation		
Accreditation impact	-0.163 (0.084)	0.055
Staff involvement	0.309 (0.105)	***0.004*** **
Accreditation awareness	0.036 (0.063)	0.569

*The model was controlled for gender, experience at the center, and position.

**Bold and italic formatting indicates significant p-values.

### Qualitative results

Out of the 25 directors that were approached, 22 directors participated in the semi-structured interviews. By counting the number of times each theme was cited during the interviews, we identified the percentage of responses related to each theme. The subsequent section summarizes results of thematic analysis (Table 5).

**Table 5:** Thematic analysis of the semi-structured interviews

**Topic**	N (%)*
**Benefits of accreditation**	
Documentation	12(55%)
Recording minutes of meetings	
Thoroughly completing medical records	
Documenting rules and regulations	
Translation of theories of quality into actions	9(41%)
Introduction and reinforcement of quality standards	7(32%)
Infection control	
Occupational safety	
Waste management	
Fire management	
Incident and accident reporting	
Enhanced employee awareness and involvement	7(32%)
Giving guidance to employees	
Empowering employees and engaging them in decision making	
Developing a job description for employees and clarifying their tasks	
Better evaluation of employees	
Better relationship between the centers and the communities they serves	5(23%)
Role of social workers	
Health awareness lectures and campaigns	
Community needs assessment	
Home visits	
Improved work conditions	4(18%)
Work flow became more organized and systematic	
Enhanced role of management and leadership	3(14%)
Forming interdisciplinary quality team	
Strategic plans	
Action plans	
Better relationship between the centers and patients	3(14%)
Follow-up on patients	
Taking client suggestions, complaints and compliments into consideration	
Enhanced patient confidentiality	
Better relationship between the centers and local authorities	2(9%)
Strengthened relationship with the Ministry of Public Health	
Strengthened relationship with the Ministry of Social Affairs	
Strengthened relationship with municipalities	
**The effect of accreditation on staff**	
Staff training, education and development	10(45%)
Staff perceived accreditation as an opportunity to develop themselves	
Staff perceived accreditation as an opportunity to help the society	
Accreditation made staff more aware about their rights	
Enhanced communication between staff and the management	3(14%)
Engaging staff from the beginning of the process	
Allowing staff to voice their opinions and concerns regarding accreditation	
Enhanced communication among staff	3(14%)
The importance of teamwork was emphasized	
**The effect of accreditation on patients**	
Increased patient satisfaction	8(36%)
Increased satisfaction with the setting	
Increased satisfaction with sanitation	
Increased satisfaction with the quality of services	
Increased patient trust in the center	
Number of patients increased	7(32%)
Attracting more patients from neighboring villages and higher social class	
Enhanced relationship between patients and the medical team	4(18%)
Physicians compliance to appointments	
Nurses involvement in patient care	
**Enabling and success factors**	
Commitment and support from the management	7(32%)
Forming an accreditation committee	
Distributing tasks	
Good teamwork	
Continuous training sessions and workshops	7(32%)
**Challenges**	
Financial barriers	11(50%)
Staff resistance	11(50%)
Accreditation was a new and vague concept	
Difficulty in communicating the importance of accreditation	
Resistance more prevalent among older employees	
Staff shortages	8(36%)
Heavy workload	
Not able to ensure enough physicians and specialists	
High turnover rate of staff	
Physicians have limited time to assess medical history and complete medical record	
Not all the standards are applicable to the context of PHC centers in Lebanon	7(32%)
Referral system among centers and to hospitals is lacking	3(14%)
**Strategies for improving implementation of accreditation**	
Financial support	10(45%)
From Ministry of Public Health and international agencies	
Follow-up meetings and communication and collaboration with the MOPH, the accreditation team, and among PHC centers, and hospitals	11(50%)
Local experts are recommended to perform assessment	4(18%)
Practical training sessions and continuing education	2(9%)
Engaging municipalities to gain their support	2(9%)

*Percentages are calculated out of a total of 22 facility directors who responded to the interview.

### Benefits of accreditation

All directors affirmed that accreditation has led to quality improvement in several areas, particularly in documentation (55% of directors) including recording minutes of meetings, thoroughly completing medical records and documenting rules and regulations.

Another frequently mentioned benefit of accreditation was translating theories of quality into action (41%). For example, implementing standards, policies and procedures and rules and regulations provided a method for centers to translate their mission, vision and values. In addition, by introducing new quality standards and reinforcing existing ones, such as infection control, occupational safety, waste and fire management, and incident and accident reporting, centers were able to translate the notions of quality into tangible outcomes, which can be measured and compared with other centers nationally and internationally (32%).

Furthermore, accreditation was perceived to enhance the awareness and involvement of employees in quality issues (32%) by guiding employees in performing their tasks and empowering them to engage in decision-making.

Other mentioned benefits of accreditation included: strengthened relationships between the centers and the communities they serve (23%), improved work conditions (18%), enhanced management and leadership (14%), and strengthened relationships between the centers and patients (14%) and local authorities (9%).

### The effect of accreditation on staff and patient satisfaction

Directors indicated that training and education was provided to staff to better prepare them for the accreditation process, which helped employees perceive accreditation as an opportunity for professional development and for providing high-quality services (45%). Accreditation helped enhance communication and teamwork among staff (14%) and between staff and management (14%).

With regards to patient satisfaction, accreditation was associated with an increase in patient trust and satisfaction with the setting and the quality of services and a decrease in the number of concerns and complaints (36%). Directors reported that the number of patients visiting the center increased (32%). Importantly, the centers attracted people from neighboring villages and higher social class, as one director reported:

*“The number of patients increased and the same patients kept coming back (…) the patients are now bringing their family members and this means that we could reach the community in a better way”*.

The increase in the number of patients might be due to the strengthened relationship between patients and the medical team (18%), as one director illustrated:

“Patients started feeling that nurses are better communicating with them and explaining to them what they need to know instead of leaving them in the dark. Patients felt that the medical team was more involved in their healthcare”.

### Enabling factors and challenges to the process of accreditation

The commitment and support from management, establishing an accreditation committee to guide implementation, distributing tasks and effective teamwork (32%), as well as continuous training and workshops were reported as key for implementing a smoother accreditation process (32%).

At the same time, facility directors reported facing many challenges in implementing accreditation. Limited financial resources were the main challenge impeding the implementation of the standards (50%). For example, financial resources were needed for waste incinerators, follow-up calls, infrastructure, equipment and information technology.

Resistance from staff, especially among older staff and physicians, was also reported as a major challenge (50%). Staff initially perceived accreditation as vague concept and were anxious about being surveyed and about the increased amount of work. However, the extensive workshops conducted by the MOPH to introduce staff members to concepts of quality and accreditation were successful in overcoming this challenge.

The limited availability of physicians and specialists and the high turnover and workload were reported to hinder the accreditation process and affect the quality of services provided (36%). Additionally, some directors reported that some accreditation standards are not fully applicable to the context of PHC centers in Lebanon (32%), as one director indicated:

*“Accreditation standards should be tailored to the Lebanese context so that we can be more compliant to them”*.

The lack of communication and collaboration between centers and the absence of a referral system among centers and to hospitals was another frequently reported challenge to accreditation (14%).

### Strategies to for improving implementation of accreditation

The need for financial support was repeatedly suggested for improving the implementation of accreditation (45%), as two directors illustrated:

“[Introducing PHC accreditation was] like providing a system that works on electricity but not providing electricity to allow functioning of these services”.

“[Financial support was needed to employ staff] for monitoring, supervising, and guiding the continuous implementation of improvements in order to maintain sustainability of the developed measures, and complete the development and implementation of all measures”.

Directors suggested targeting the MOPH and international agencies for sources of funding.

Conducting follow-up meetings and communication and collaboration with the MOPH, the accreditation team, and among PHC centers, and hospitals were suggested for sharing experiences on implementing accreditation (50%). Some directors suggested that local experts should be involved in conducting the accreditation survey and the financial resources that would otherwise be used to employ foreign surveyors could be invested in improving the delivery of services (18%), as one director suggested:

*“[One strategy to improve implementation is to] establish a voluntary national committee that includes experts from Lebanon [*who are aware of the context of PHC centers in Lebanon] *in order to conduct accreditation surveys”*.

## Discussion

This study demonstrated the positive impact of accreditation on PHC centers in several areas of quality and performance. Accreditation was associated with improved delivery of health care and quality. For example, significant improvements were reported for documentation, which initially was a challenging area for most PHC centers [16]. Improvements in quality were reflected by the increase in customer satisfaction and number of patients visiting PHC centers from various regions and social strata.

Regression findings revealed that strategic quality planning, customer satisfaction and staff involvement were associated with better scores on quality results. Accreditation has been linked with improved staff satisfaction of quality management and planning and has been linked to leadership styles particularly those that are open to organizational development [7]. An indirect outcome of accreditation is improving patient satisfaction. Accreditation has been linked to improved client satisfaction in previous related research particularly in terms of addressing user complaints [7]. In terms of staff involvement, results demonstrated a link between this subscale and quality results. Moreover, the increase in staff involvement in accreditation helped enhance their professional development and awareness in quality issues and encouraged them to voice their opinions, which in turn might have helped improve quality results. At the same time, findings showed that staff were not often rewarded or recognized for their efforts, which might influence their satisfaction and the sustainability of improvements. An earlier study focusing on the impact of accreditation in hospitals in Lebanon found a significant association between quality results and staff involvement [13]. Another study found that staff who were not involved in the satisfaction process could not perceive the benefits of this process on their healthcare organization [7]. This comes to further highlight the importance of staff involvement when implementing new initiatives in health organizations and it also specific reduces resistance to change [23].

Strong leadership and support from management were critical for implementing accreditation and improving quality of services in PHC centers. Additionally, training of staff members was essential for overcoming resistance.

Limited financial resources impeded PHC centers from recruiting specialized staff and purchasing equipment to enable implementation of some accreditation standards. The ministry’s contracting transactions with centers does not involve money transactions, but involves training and the provision of vaccines, essential drugs and medical and educational supplies. Insufficient resources and staff, unpredictable drug supplies and faulty equipment are some key challenges facing PHC worldwide. Furthermore, high staff turnover and workload, and the absence of a referral system were major challenges to implementing some of the PHC accreditation standards.

### Findings in relation to other studies

Similar to this study, findings from El-Jardali et al. (2008) on the impact of accreditation on hospitals in Lebanon showed that accreditation improved quality of care and that staff involvement helped improve quality results [13]. A systematic review on health sector accreditation also reported increased professional development [24].

Challenges reported in this study parallel those reported in other studies [13,16,24-27]. A period of high workload and job stress was previously reported to accompany hospital accreditation [26,27]. Furthermore, a systematic review on health sector accreditation showed that the costs of accreditation were high for individual organizations and questioned whether accreditation was an appropriate use of resources [24]. Whereas another study concluded that the costs incurred in participating in accreditation should be viewed as an essential investment [28].

Findings on key enablers for implementing accreditation confirmed those reported previously. Leadership and commitment for hospital accreditation were previously identified as predictors of improved quality results [13] and were considered major components for the successful implementation of accreditation [16,29]. Early and frequent communication with all stakeholders (individual facilities, local authorities, NGOs) for sharing experiences was also a major element for successful implementation [29,30]. Education and training of staff were critical for the implementation of accreditation [9,13,16]. Additionally, providing incentives, resources, rewards and publicizing the names of centers were considered effective marketing tools for the centers [31].

### Strengths and limitations

This study is among the very few studies in the region (if not the first), and the first in Lebanon to assess the impact of accreditation on the quality of services provided in the PHC setting. The study utilized a mixed methods approach for data collection, which combined the benefits of quantitative and qualitative approaches to strengthen the validity of results.

Some limitations to this study should be acknowledged. The results are based on the perception of PHC staff, with no further analysis of patient outcome data. However, patient outcome data are not readily available or accessible at PHC centers. It is highly recommended that future research examine the impact of accreditation in conjunction with patient outcome measures. Another limitation is social desirability bias inherent with self-reported questionnaires. Respondents from PHC centers may have provided answers they considered desirable to the researchers. However, it can be safely assumed that the results are not overly inflated because of the positive nature of almost all the questions. Another limitation of the survey was that we cannot accurately assess the different between respondents and non-respondents. The centers included in this survey all underwent the accreditation survey. The MOPH is current working on building capacity of surveyors and PHC center staff in preparation for another accreditation survey. It is hoped that this study can then be replicated on a larger scale.

Finally, it should be noted that this study followed a cross sectional design. These studies are often described as taking a “snapshot” of a particular population. While they have limited values in testing hypotheses, they can be useful in assessing practices, attitudes, knowledge and beliefs. Results of cross sectional studies can give an indication of the magnitude of the condition/problem and help researchers obtain information on patterns and trends which can help them establish proper interventions. However, cross sectional studies cannot ascertain causality [32].

## Conclusion

Having high quality primary health care system leads to better health outcomes. This study emphasizes the importance of accreditation as a first step towards improving the quality of PHC delivery arrangement system. PHC in Lebanon has a potential to expand coverage and improve outcomes. However, to achieve these goals, all PHC centers in Lebanon need to implement accreditation and receive support both in financial and non-financial resources. This will help strengthen the financial and delivery arrangements of PHC. It is recommended that the MOPH in Lebanon to expand the implementation of accreditation to cover all PHC centers and to explore several options to better finance PHC including contracting out (by the MOPH) for the delivery of PHC using capitation; subsidizing individual enrollment fees in PHC or purchasing of PHC services by third party payers, etc. Accreditation standards should also be revised regularly to ensure that they comply with most recent worldwide standards and continue to address the context of Lebanon. There is also a need to build the capacity of PHC centers to allow them to better comply with accreditation standards.

Strengthening the delivery arrangement of PHC should be associated with strengthening its financial arrangements. Findings from this study provide important lessons for improving the implementation of accreditation in Lebanon specifically and for other countries from the region and beyond that are currently implementing or planning to implement accreditation.

## Competing interests

The authors declare that they have no competing interests.

## Authors’ contributions

FE contributed to the conception, study design, tool development, data collection, as well as data analysis and interpretation of results in addition to development of the manuscript. RH contributed to data collection, and interpretation of results. MJ contributed to tool development, data collection, data analysis and manuscript preparation. LS, RE and RM contributed to conception, study design, tool development, data collection and data entry. DJ contributed to data analysis and interpretation and manuscript preparation. NA contributed to manuscript preparation and review. All authors read and approved the final manuscript.

## Pre-publication history

The pre-publication history for this paper can be accessed here:

http://www.biomedcentral.com/1472-6963/14/86/prepub

## Supplementary Material

Additional file 1 Detailed responses to survey questions. Click here for file

## References

[B1] Australian Government, Department of Health and Ageing Primary health care reform in Australia: Report to Support Australia’s First National Primary Health Care Strategy 2009 Australia Available at: http://www.yourhealth.gov.au/internet/yourhealth/publishing.nsf/Content/nphc-draftreportsupp-toc/$FILE/NPHC-supp.pdf. Last accessed: January 22, 2012

[B2] Russell G Geneau R Johnston S Liddy C Hogg W Hogan K Mapping the future of primary healthcare research in Canada: a report to the Canadian Health Services Research Foundation 2009 Canadian Health Services Research Available at: http://www.chsrf.ca/migrated/pdf/researchReports/commissionedResearch/mapping_future_report_2007_e.pdf. Last accessed: January 22, 2012

[B3] Schoen C Osborn R Doty MM Squires D Peugh J Applebaum S A survey of primary care physicians in eleven countries, 2009: perspectives on care, costs, and experiences Health Aff 2009 14 6 w1171 w1183 10.1377/hlthaff.28.6.w1171 19884491

[B4] World Health Organization The World Health Report. Primary healthcare: now more than ever 2008 Geneva, Switzerland: WHO

[B5] World Health Organization Quality improvement in primary health care: a practical guide 2004 Geneva, Switzerland: WHO

[B6] Pomey MP Lemieux-Charles L Champagne F Angus D Shabah A Contandriopoulos AP Does accreditation stimulate change? A study of the impact of the accreditation process on Canadian healthcare organizations Implement Sci 2010 14 31 10.1186/1748-5908-5-31 20420685 PMC2882897

[B7] Paccioni A Sicotte C Champagne F Accreditation: a cultural control strategy Int J Health Care Qual Assur 2008 14 2 146 158 10.1108/09526860810859012 18578200

[B8] World Health Organization Quality and accreditation in health care services: a global review 2003 Geneva, Switzerland: WHO

[B9] Buetow SA Wellingham J Accreditation of general practices: challenges and lessons Qual Saf Health Care 2003 14 129 135 10.1136/qhc.12.2.129 12679510 PMC1743687

[B10] O'Beirne M Zwicker K Sterling PD Lait J Lee Robertson H Oeike ND The status of accreditation in primary care Qual Prim Care 2013 14 23 31 23735631

[B11] World Health Organization-Regional Office for the Eastern Mediterranean Expert group meeting on hospital accreditation 2002 Cairo, Egypt: WHO EMRO

[B12] El-Jardali F Hospital accreditation in Lebanon: its potential for quality improvement Lebanese Med J 2007 14 39 45 17489306

[B13] El-Jardali F Jamal D Dimassi H Ammar W Tchaghchghian V The impact of hospital accreditation on quality of care: perception of Lebanese nurses Int J Qual Health Care 2008 14 363 371 18596050 10.1093/intqhc/mzn023

[B14] Ammar W Health Beyond Politics 2009 Beirut: World Health Organization, Eastern Mediterranean Regional Office

[B15] Hemadeh R The national primary healthcare strategy 2013 Beirut, Lebanon: Ministry of Public Health

[B16] El-Jardali F Ammar W Hemadeh R Jamal D Jaafar M Improving Primary Health Care Through Accreditation: Baseline Assessment of Readiness and Challenges in Lebanese Context In Press 10.1002/hpm.2170 23512306

[B17] Accreditation Canada International Lebanon Primary Care Standards 2011 ACI

[B18] Audette S Enabling quality improvement within primary care 2011 Medhealth Health Management Forum, Accreditation Canada International

[B19] Mohamad Ali A El Jardali F Kassak K Ramadan S Harnessing the private sector to achieve public health goals in counties of the Eastern Mediterranean: Focus on Lebanon 2005 Beirut: Department of Health Management and Policy, Faculty of Health Sciences, American University of Beirut

[B20] Milner BM Implementing hospital accreditation: individual experiences of process and impacts 2007 Thesis submitted to Higher Education Training Awards Council in fulfillment of the degree of doctor of philosophy to the Waterford Institute of Technology, School of Business

[B21] Hughes KA Comparing Pretesting Methods: Cognitive Interviews, Respondent Debriefing, and Behavior Coding 2004 Washington D.C: US Bureau of the Census

[B22] Willis GB Cognitive Interviewing A “How To” Guide 1999 Research Triangle Institute Available on: http://www.hkr.se/pagefiles/35002/gordonwillis.pdf

[B23] Seren S Baykal U Relationships between change and organizational culture in hospitals J Nurs Scholarsh 2007 14 181 187 10.1111/j.1547-5069.2007.00166.x 17535321

[B24] Greenfield D Braithwaite J Health sector accreditation research: a systematic review Int J Qual Health Care 2008 14 3 172 183 10.1093/intqhc/mzn005 18339666

[B25] Greenfield D Pawsey M Naylor J Braithwaite J Are healthcare accreditation surveys reliable? Int J Health Care Qual Assur 2009 14 2 105 116 10.1108/09526860910944601 19536962

[B26] Manzo BF Brito MJ Corrêa Ados R Implications of hospital accreditation on the everyday lives of healthcare professionals Rev Esc Enferm USP 2012 14 2 388 394 10.1590/S0080-62342012000200017 22576543

[B27] Elkins G Cook T Dove J Markova D Marcus JD Meyer T Rajab MH Perfect M Perceived stress among nursing and administration staff related to accreditation Clin Nurs Res 2010 14 4 376 386 doi:10.1177/1054773810373078 10.1177/1054773810373078 20601637 PMC3907189

[B28] Mihalik G Scherer M Schreter R The high price of quality: a cost analysis of NCQA accreditation J Health Care Finance 2003 14 38 47 12635993

[B29] Woodcock S Fine G McClure K Unger B Rizzo-Price P The role of standards and training in preparing for accreditation Am J Clin Pathol 2010 14 3 388 392 doi:10.1309/AJCP03TFPBKEYYNT 10.1309/AJCP03TFPBKEYYNT 20716794

[B30] Cleveland EC Dahn BT Lincoln TM Safer M Podesta M Bradley E Introducing health facility accreditation in Liberia Glob Publ Health 2011 14 3 271 282 doi:10.1080/17441692.2010.489052 10.1080/17441692.2010.489052 PMC3062234 20623390

[B31] Alkhenizan A Shaw C The attitude of health care professionals towards accreditation: a systematic review of the literature J Fam Community Med 2012 14 2 74 80 doi:10.4103/2230-8229.98281 10.4103/2230-8229.98281 PMC3410183 22870409

[B32] Bryman A Social Research Methods 2012 4 New York: Oxford University Press

