# Supplementary material for: The impact of accreditation of primary healthcare centers: successes, challenges and policy implications as perceived by healthcare providers and directors in Lebanon
Source: BMC Health Serv Res. 2014 Feb 25;14:86. doi: 10.1186/1472-6963-14-86 (PMC3946059; doi:10.1186/1472-6963-14-86)
Supplement: Additional file 1 — Detailed responses to survey questions. [file 1472-6963-14-86-S1.docx]

**Appendix I**

**Responses on Management and Leadership, Strategic Quality Planning and Quality Management questions**

|  | **Strongly disagree/ Disagree** | **Neither disagree nor agree** | **Strongly agree/ Agree** |
| --- | --- | --- | --- |
|  | **N (%)** | **N (%)** | **N (%)** |
| **Management and Leadership** | | | |
| 1. The senior executives provide highly visible leadership in maintaining an environment that supports quality improvement. | 5 (1.7%) | 7 (2.3%) | 291 (96%) |
| 1. The top management is a primary driving force behind quality improvement efforts. | 4 (1.3%) | 8 (2.6%) | 291 (96%) |
| 1. The senior executives allocate available resources (e.g. finances, people, time, equipment) to improving quality. | 5 (1.7%) | 14 (4.7%) | 280 (93.6%) |
| 1. The senior executives consistently participate in activities to improve the quality of care and services. | 5 (1.7%) | 15 (5%) | 281 (93.4%) |
| 1. The senior executives have articulated a clear vision for improving the quality of care and services. | 6 (2.1%) | 17 (5.8%) | 269 (92.1%) |
| 1. The senior executives have demonstrated an ability to manage the changes (e.g. organizational, technological) needed to improve the quality of care and services. | 4 (1.4%) | 23 (7.8%) | 266 (90.8%) |
| 1. The senior executives started to act on suggestions to improve the quality of care and services. | 4 (1.3%) | 14 (4.7%) | 281 (94%) |
| 1. Based on the accreditation results, senior executives have a thorough understanding of how to improve the quality of care and services. | 4 (1.3%) | 20 (6.6%) | 277 (92%) |
| 1. The senior executives generate confidence that efforts to improve quality will succeed. | 1 (0.3%) | 15 (5%) | 282 (94.6%) |
| **Strategic Quality Planning** | | | |
| 1. Staff members are given adequate time to plan for and test quality improvements. | 12 (4.2%) | 37 (12.8%) | 240 (83%) |
| 1. Each department and work group within this center maintains specific goals to improve quality. | 3 (1%) | 16 (5.3%) | 285 (93.8%) |
| 1. The center's quality improvement goals are known throughout your unit. | 4 (1.3%) | 14 (4.6%) | 287 (94.1%) |
| 1. Staff members are involved in developing plans for improving quality. | 8 (2.7%) | 24 (8.1%) | 265 (89.2%) |
| 1. Middle managers (e.g. Nurse Heads, Director of Nursing, Clinical specialists) play a key role in setting priorities for quality improvement. | 8 (2.7%) | 19 (6.3%) | 273 (91%) |
| 1. Patients’ expectations about quality play a key role in setting priorities for quality improvement. | 13 (4.5%) | 33 (11.5%) | 240 (83.9%) |
| 1. Staff members play a key role in setting priorities for quality improvement through representation in the center’s organizational chart. | 5 (1.7%) | 10 (3.3%) | 287 (95%) |
| **Quality Management** | | | |
| 1. The center regularly checks equipment and supplies to make sure they meet quality requirements. | 8 (2.7%) | 10 (3.3%) | 281 (94%) |
| 1. The center has effective policies to support improving the quality of care and services | 8 (2.7%) | 13 (4.4%) | 276 (92.9%) |
| 1. The center tries to design quality into new services as they are being developed. | 5 (1.7%) | 20 (6.8%) | 270 (91.5%) |
| 1. The services that the center provides are thoroughly tested for quality before they are implemented. | 8 (2.8%) | 19 (6.6%) | 259 (90.6%) |
| 1. The center views quality assurance as a continuing search for ways to improve. | 6 (2%) | 13 (4.4%) | 277 (93.6%) |
| 1. The center encourages staff members to keep records of quality problems through documentation. | 7 (2.4%) | 17 (5.9%) | 266 (91.7%) |
| **Human Resources Utilization** |  |  |  |
| 1. Staff members are given education and training in how to identify and act on quality improvement opportunities based on recommendations from accreditation surveys. | 8 (2.7%) | 17 (5.8%) | 267 (91.4%) |
| 1. Staff members are given continuous education and training in methods that support quality improvement. | 10 (3.4%) | 21 (7.1%) | 266 (89.6%) |
| 1. Staff members are given the needed education and training (through education programs) to improve job skills and performance. | 10 (3.4%) | 28 (9.6%) | 255 (87%) |
| 1. Staff members are rewarded and recognized (e.g. financially and/or otherwise) for improving quality. | 53 (19.7%) | 48 (17.8%) | 168 (62.5%) |
| 1. Inter-departmental cooperation to improve the quality of services is supported and encouraged. | 7 (2.3%) | 28 (9.4%) | 263 (88.3%) |
| 1. The center has an effective system for staff members to make suggestions to management on how to improve quality. | 22 (7.5%) | 20 (6.8%) | 250 (85.6%) |
| **Quality Results** |  |  |  |
| 1. Over the past year, the center has shown steady, measurable improvements in the quality of customer satisfaction. | 5 (1.7%) | 21 (7.3%) | 263 (91%) |
| 1. Over the past year, the center has shown steady, measurable improvements in the quality of services provided by the administration (e.g. finance, human resources. | 9 (3.2%) | 25 (9%) | 244 (87.8%) |
| 1. Over the past year, the center has shown steady, measurable improvements in the quality of care provided to patients (e.g. medical, surgical, obstetric, paediatric patients). | 7 (2.5%) | 25 (8.9%) | 250 (88.7%) |
| 1. Over the past year, the center has shown steady, measurable improvements in the quality of services provided by clinical support departments (e.g. laboratory, pharmacy, radiology). | 1 (0.4%) | 26 (9.2%) | 256 (90.5%) |
| 1. Over the past year, the center has maintained a high quality health services despite financial constraints. | 3 (1%) | 14 (4.8%) | 274 (94.2%) |
| **Customer (Patient) Satisfaction** |  |  |  |
| 1. The center does a good job of assessing current patient needs and expectations. | 3 (1%) | 13 (4.4%) | 282 (94.6%) |
| 1. The center does a good job of assessing future patient needs and expectations. | 5 (1.7%) | 21 (7.2%) | 267 (91.1%) |
| 1. Staff members promptly resolve patient complaints. | 4 (1.3%) | 17 (5.7%) | 279 (93%) |
| 1. Patients' complaints are studied to identify patterns and learn from them to prevent the same problems from recurring. | 8 (2.7%) | 25 (8.5%) | 261 (88.8%) |
| 1. The center uses data from patients to improve services. | 3 (1%) | 29 (10%) | 257 (88.9%) |
| 1. Data on patient satisfaction are widely communicated to staff members. | 15 (5.5%) | 31 (11.4%) | 225 (83%) |
| 1. The center uses data on patient expectations and/or satisfaction when designing new services. | 6 (2.2%) | 25 (9.1%) | 245 (88.8%) |
| **Accreditation Impact** |  |  |  |
| 1. During the preparation for the last survey, important changes were implemented at the center. | 7 (2.4%) | 24 (8.2%) | 260 (89.3%) |
| 2. You participated in the implementation of these changes. | 14 (4.7%) | 18 (6%) | 268 (89.3%) |
| 3. You learned of the recommendations made to your center since the last survey (if it’s the case). | 11 (3.8%) | 11 (3.8%) | 271 (92.5%) |
| 4. These recommendations were an opportunity to implement important changes at the center. | 10 (3.4%) | 18 (6.2%) | 262 (90.3%) |
| 5. You participated in the changes that resulted from accreditation recommendations. | 10 (3.4%) | 23 (7.8%) | 261 (88.8%) |
| 6. Accreditation enables the improvement of patient care. | 4 (1.4%) | 22 (7.5%) | 268 (91.2%) |
| 7. Accreditation enables the motivation of staff and encourages team work and collaboration | 1 (0.3%) | 14 (4.8%) | 278 (94.9%) |
| 8. Accreditation enables the development of values shared by all professionals at the center. | 2 (0.7%) | 23 (7.9%) | 265 (91.4%) |
| 9. Accreditation enables the center to better use its internal resources (e.g. finances, people, time, equipment). | 4 (1.4%) | 22 (7.6%) | 262 (91%) |
| 10. Accreditation enables the center to better respond to the populations needs. | 5 (1.8%) | 34 (12%) | 245 (86.3%) |
| 11. Accreditation enables the center to better respond to its partners (e.g. other centers, diverse hospitals, private clinics, etc.) | 9 (3.3%) | 39 (14.4%) | 223 (82.3%) |
| 12. Accreditation contributes to the development of collaboration with partners in the health care system. | 2 (0.7%) | 19 (6.7%) | 264 (92.6%) |
| 13. Accreditation is a valuable tool for the center to implement changes. | (%) | 17 (5.7%) | 282 (94.3%) |
| 14. The center’s participation in accreditation enables it to be more responsive when changes are to be implemented. | 1 (0.3%) | 15 (5.1%) | 281 (94.6%) |
| **Staff Involvement in the Accreditation Process** |  |  |  |
| 1. I received sufficient training and support in order to fulfill my accreditation responsibilities. | 16 (5.4%) | 49 (16.4%) | 233 (78.2%) |
| 1. There was sufficient leadership for the accreditation process. | 14 (4.7%) | 36 (12.1%) | 247 (83.2%) |
| 1. The overall accreditation process was well managed. | 5 (1.7%) | 21 (7.2%) | 267 (91.1%) |
| 1. Our team worked well together. | 6 (2%) | 19 (6.4%) | 273 (91.6%) |
| 1. Everyone was encouraged to participate in the accreditation process. | 2 (0.7%) | 15 (5%) | 281 (94.3%) |
| 1. Everyone had the opportunity to voice their opinions. | 3 (1%) | 34 (11.4%) | 261 (87.6%) |
| 1. I felt part of an accreditation team. | 5 (1.7%) | 22 (7.3%) | 273 (91%) |
| 1. Staff members took the agreed deadlines seriously. | 3 (1%) | 11 (3.8%) | 279 (95.2%) |
| 1. I was fully committed to accreditation at all stages of the process. | 6 (2%) | 20 (6.7%) | 272 (91.3%) |
| 1. Accreditation enhanced my relationships with my immediate work colleagues. | 7 (2.4%) | 37 (12.5%) | 252 (85.1%) |
| 1. My work colleagues assisted and supported me in completing my accreditation tasks. | 11 (3.7%) | 30 (10.2%) | 253 (86.1%) |
| 1. My line manager assisted and supported me in completing my accreditation tasks. | 8 (2.7%) | 31 (10.4%) | 259 (86.9%) |
| 1. I got recognition from my work colleagues for my contribution to the accreditation process. | 23 (8.1%) | 49 (17.2%) | 213 (74.7%) |
| 1. I got recognition from my line manager for my contribution to the accreditation process. | 25 (8.7%) | 39 (13.5%) | 225 (77.9%) |
| 1. Involvement in the accreditation process has allowed me to reflect on my work practices. | 10 (3.4%) | 22 (7.4%) | 266 (89.3%) |
| 1. Involvement in the accreditation process contributed to my personal development. | 19 (6.5%) | 37 (12.6%) | 238 (81%) |
| 1. Involvement in the accreditation process contributed to my professional development. | 13 (4.3%) | 36 (12%) | 252 (83.7%) |
| 1. Involvement in the accreditation process will contribute to my career advancement. | 8 (2.8%) | 28 (9.7%) | 252 (87.5%) |
| 1. Accreditation has improved the level of multidisciplinary working in the center. | 4 (1.4%) | 32 (10.8%) | 260 (87.8%) |
| 1. Accreditation has improved the standard and delivery of healthcare within my immediate work environment. | 4 (1.3%) | 24 (8.1%) | 269 (90.6%) |
| 1. Accreditation has improved the standard and delivery of healthcare within the center. | 3 (1%) | 21 (7.1%) | 273 (91.9%) |
| 1. Accreditation is a worthwhile process. | 2 (0.7%) | 6 (2%) | 294 (97.4%) |
| **Awareness of the Accreditation Process** |  |  |  |
| 1. Staff members in the center are aware that the accreditation process is taking place. | 2 (0.7%) | 9 (3%) | 289 (96.3%) |
| 1. Staff members in the center are aware of the aims and objectives of the accreditation process. | 3 (1%) | 16 (5.4%) | 280 (93.6%) |
| 1. Staff members in the center believe that accreditation is a worthwhile process. | 3 (1%) | 15 (5.2%) | 271 (93.8%) |
| 1. Patients are aware that the accreditation process is underway. | 20 (7.5%) | 59 (22%) | 189 (70.5%) |
| 1. Other associated healthcare organizations in the region are aware that the accreditation process in the center is underway. | 9 (3.9%) | 41 (17.7%) | 182 (78.4%) |
